# Superparamagnetic iron oxide nanoparticles mediated ^131^I-hVEGF siRNA inhibits hepatocellular carcinoma tumor growth in nude mice

**DOI:** 10.1186/1471-2407-14-114

**Published:** 2014-02-21

**Authors:** Jing Chen, Shu Zhu, Liangqian Tong, Jiansha Li, Fei Chen, Yunfeng Han, Ming Zhao, Wei Xiong

**Affiliations:** 1 Department of Nuclear Medicine, Tongji Hospital, Tongji Medical College, Huazhong University of Science and Technology, Wuhan, Hubei 430030, China; 2 Department of Ultrasound, Tongji Hospital, Tongji Medical College, Huazhong University of Science and Technology, Wuhan, Hubei 430030, China; 3 Department of Radiology, Haikou People’s Hospital, Xiangya Medical Cellege, Central South University, Haikou 570208, China; 4 Pathology Department, Tongji Hospital, Tongji Medical College, Huazhong University of Science and Technology, Wuhan, Hubei 430030, China; 5 Department of Orthopaedics, Tongji Hospital, Tongji Medical College, Huazhong University of Science and Technology, Wuhan, Hubei 430030, China

**Keywords:** VEGF, Small interfering RNA, ^131^I, Hepatocellular carcinoma, SPIOs

## Abstract

**Background:**

Hepatocellular carcinoma (HCC) is a primary liver tumor and is the most difficult human malignancy to treat. In this study, we sought to develop an integrative approach in which real-time tumor monitoring, gene therapy, and internal radiotherapy can be performed simultaneously. This was achieved through targeting HCC with superparamagnetic iron oxide nanoparticles (SPIOs) carrying small interfering RNA with radiolabled iodine 131 (^131^I) against the human vascular endothelial growth factor (hVEGF).

**Methods:**

hVEGF siRNA was labeled with ^131^I by the Bolton-Hunter method and conjugated to SilenceMag, a type of SPIOs. ^131^I-hVEGF siRNA/SilenceMag was then subcutaneously injected into nude mice with HCC tumors exposed to an external magnetic field (EMF). The biodistribution and cytotoxicity of ^131^I-hVEGF siRNA/SilenceMag was assessed by SPECT (Single-Photon Emission Computed Tomography) and MRI (Magnetic Resonance Imaging) studies and blood kinetics analysis. The body weight and tumor size of nude mice bearing HCC were measured daily for the 4-week duration of the experiment.

**Results:**

^131^I-hVEGF siRNA/SilenceMag was successfully labeled; with a satisfactory radiochemical purity (>80%) and biological activity in vitro. External application of an EMF successfully attracted and retained more ^131^I-hVEGF siRNA/SilenceMag in HCC tumors as shown by SPECT, MRI and biodistribution studies. The tumors treated with ^131^I-hVEGF siRNA/SilenceMag grew nearly 50% slower in the presence of EMF than those without EMF and the control. Immunohistochemical assay confirmed that the tumor targeted by ^131^I-hVEGF siRNA/SilenceMag guided by an EMF had a lower VEGF protein level compared to that without EMF exposure and the control.

**Conclusions:**

EMF-guided ^131^I-hVEGF siRNA/SilenceMag exhibited an antitumor effect. The synergic therapy of ^131^I-hVEGF siRNA/SilenceMag might be a promising future treatment option against HCC with the dual functional properties of tumor therapy and imaging.

## Background

HCC is the fifth most common cancer and the third leading cause of cancer-related death in the world, with a particularly high prevalence in Asia and an increasing incidences in North America, Western Europe and Japan [1]. Although surgical resection and liver transplantation are potential curative treatments for HCC, their applications are limited by a high incidence of postoperative recurrence and the shortage of grafts. Alternative strategies such as TACE (TransArterial ChemoEmbolization) have been applied without showing any improvement in the median survival of patients [2]. Conventional chemotherapy has been proven insensitive for HCC [3]. To date, no systemic treatments in HCC offered any significant benefit to patients [4]. Thus, the search for new therapeutic strategies for HCC is of paramount importance.

HCC is known as a hypervascularized tumor expressing extensive amounts of VEGF, which correlates with progressive tumor growth [5,6]. VEGF is stated as the major factor contributing to cancer cell survival and proliferation after irradiation treatment [7-9]. Several therapeutic approaches have been developed through inhibiting the binding of VEGF to its receptors, such as soluble VEGF receptors [10] or anti VEGF antibodies [11]. The disadvantage of these approaches is that they were unable to capture or neutralize the VEGF protein that is already circulating in the system. In this regard, VEGF siRNA is considered as a better approach for more complete knocking down of VEGF. SiRNA targeted against VEGF has been shown to silence the VEGF gene, to reduce the levels of VEGF proteins, and to inhibit tumor growth in HCC models [12,13]. However, efficient delivery of the siRNA still remains a challenge. For example, delivery of VEGF siRNA by adenoviral vectors has off-target effect and triggers receptor-dependent host tropism. Retroviral vectors have low titers and liposome vectors show low efficiencies. To overcome these deficiencies, we conjugated siRNA vectors with SilenceMag and achieved gene delivery by application of an EMF. SilenceMag is a type of Superparamagnetic iron oxide nanoparticles (SPIOs) that are positively charged thus has good biocompatibility [14]. SPIOs can be easily combined with negatively charged materials, such as anticancer drugs and nucleic acids, which contribute greatly to targeted drug delivery and gene transduction [4,15]. SPIOs have quickly become a popular tool in cancer treatment and molecular imaging [14].

Multiple molecular imaging modalities have been applied to track the delivery of siRNAs *in vivo*. One such technique is optical imaging technique that presents several potential advantages [16,17]. However, a significant disadvantage is that imaging depth in biological tissue is limited due to the absorption and scattering of light. ^131^I was chosen for this study because its decay emits both γ rays and β particles. The γ ray was used for imaging and biodistribution studies *in vivo*, while the β particle was used for brachytherapy.

In this study, we employed superparamagnetic iron oxide nanoparticles (SPIOs) carrying human VEGF siRNA (hVEGF siRNA) labeled with iodine 131 (^131^I) and sought to test its antitumor effect in nude mice.

## Methods

### Preparation of hVEGF siRNA with 2′-O-methyl modification

**Figure 1:**
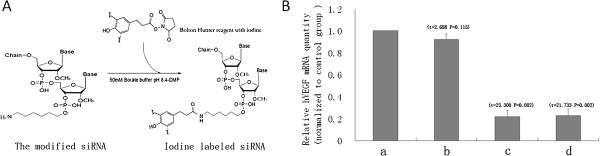
**hVEGF siRNA/SilenceMag and **^**131**^**I-hVEGF siRNA/SilenceMag knocked down hVEGF in HepG2 cells*. *A**: Scheme showing the modification of hVEGF siRNA and the Iodination of the hVEGF siRNA using Bolton-Hunter Reagent. **B**: qRT-PCR analysis of the relative hVEGF mRNA levels in HepG2 cells, **a**, blank controls; **b**, transfected with scrambled siRNA/SilenceMag; **c**, transfected with hVEGF siRNA/SilenceMag; **d**, transfected with ^131^I-hVEGF siRNA/SilenceMag.

We selected siRNA sequences as reported by Yoshifumi Takei [7]. The hVEGF siRNA (bases 189–207) with the following sense and antisense sequences was used: 5′-GGAGUACCCUGAUGAGAUCdTdT-3′ (sense), 5′-GAUCUCAUCAGGGUACUCCdTdT-3′ (antisense). The scrambled siRNA (SiScr) was used for a control with the following sense and antisense sequences: 5′-UUCUCCGAACGUGUCACGUTdTd-3′ (sense), 5′-ACGUGACACGUUCGGAGAAdTdT-3′ (antisense). Both siRNAs were synthesized with a 2′-*O*-methyl modification of three bases at both 5′ ends by GenePharma Co., and the sense strand of the hVEGF siRNA was modified with a 5′ amino C6 linker for radioactive labeling. Additionally, a deoxythymidine overhang was added to the 3′ end of each single strand (Figure 1A). All modified siRNAs were produced by Shanghai GenePharma Co. (Shanghai, China). Chemically synthesized siRNAs were purified and analyzed with high-performance liquid chromatogramphy (HPLC). A high-performance liquid chromatography (HPLC) column (C18, 5 μm, 4.6 × 250 mm) was purchased from Agilent Technologies (Santa Clara, CA, USA).

### Preparation of ^131^I-hVEGF siRNA/SilenceMag

^131^I-Na was purchased from Beijing Atom HighTech Co., Ltd. (Beijing, China). Bolton-Hunter reagent was purchased from Thermo Scientific Pierce (Rockford, USA). Bolton-Hunter reagent was labeled with ^131^I using the chloramine-T method and then immediately extracted using a mixture of dimethylformamide (DMF) and benzene (1:20). A gentle stream of nitrogen dried the organic phase containing the labeled reagent in a glass vial. To dissolve the dried, labeled reagent, 10 μl of DMF was added. Next, 5 nmol of hVEGF siRNA in 16 μl of 50 mM borate buffer (pH 8.5) was added to the DMF solution (Figure 1A). The mixture was vortexed for a few seconds followed by incubation on ice for 2 h. An excess amount of Bolton-Hunter reagent (10,000 times the amount of hVEGF siRNA) was dissolved in 10 μl of DMF and added to the mixture. The glass vial was then incubated at 4°C overnight. The reaction mixture was purified and concentrated by ultrafiltration through a 3,000-molecular weight cutoff filter. Amicon Ultra-0.5 centrifugal filter devices were purchased from Millipore Co. (Billerica, USA). The mixture was centrifuged twice at 14,000 × *g* for 30 min with RNase-free PBS. The concentrated sample was collected from the device, and the radiochemical yield of ^131^I-hVEGF siRNA was determined.

For preparing ^131^I-hVEGF siRNA/SilenceMag, 500 ul 10 nM ^131^I-hVEGF siRNA was mixed with 1 ul ScilenceMag, a type of SPIOs. which was purchased from OZ bioscience (France). The complexes were shortly vortexed and allowed to equilibrate for 30 min at room temperature prior to analysis. The hVEGF siRNA/SilenceMag and scrambled siRNA/SilenceMag were synthesized similarly.

### Radiochemical purity of ^131^I-hVEGF siRNA/SilenceMag

The radiochemical purity of the ^131^I-hVEGF siRNA/SilenceMag was tested by instant thin-layer chromatography (ITLC). Silica gel plates and filter paper (No. 1; Hangzhou Xinhua Paper Manufactory, Hangzhou, China) were used as the stationary phase. The mobile phase for the silica gel plate was a mixture of freshly prepared acetone and normal saline (1:1) and a mixture of freshly prepared methanol and 5% ammonium acetate (1:1) for the filter paper.

### Biological activity of ^131^I- hVEGF siRNA/SilenceMag

HepG2, a human liver cancer cell line, was obtained from the Cell Bank of Type Culture Collection of Chinese Academy of Sciences. HepG2 cells were cultured in six-well plates under 5% CO_2_ at 37°C in Dulbecco’s modified Eagle’s medium (DMEM) (Invitrogen, USA). The cells was treated as follows: group A, blank controls; group B, transfected with 100 pmol scrambled siRNA/SilenceMag; group C, transfected with 100 pmol hVEGF siRNA/SilenceMag; and group D, transfected with 100 pmol ^131^I-hVEGF siRNA/SilenceMag. The cells were incubated with transfection solution for 6 h and harvested at 24 h. RNA was isolated using Trizol reagent (Invitrogen). cDNA was synthesized using M-MLV reverse transcriptase 1^st^-strand cDNA synthesis kit from Invitrogen (Carlsbad, USA). PCR was conducted using SYBR Green purchased from Toyobo Co., Ltd. (Osaka, Japan) and a PCR amplification kit purchased from Takara Biotechnology Co., Ltd. (Shiga, Japan). Amplification was performed as follows: 95°C for 1 min, followed by 40 cycles of 95, 58, and 72°C for 15 s each. The following PCR primer pairs were used for hVEGF and β-actin, respectively: 5′-CAGCTACTGCCATCCAATCG-3′ and 5′-TTGTTGTGCTGTAGGAAGCTCA-3′, and 5′-GTCCACCGCAAATGCTTCTA-3′ and 5′-TGCTGTCACCTTCACCGTTC-3′. The amount of hVEGF mRNA was measured relative to the mean critical threshold (CT) values of β-actin mRNA using the 2^-ΔΔCT^ method.

### Animal experiment

6-week-old BALB/C nude male mice were obtained from Laboratory Animal Centre of Tongji medical college of Huazhong university of science and technology. The nude mice were maintained under specific-pathogen-free conditions in the Experimental Animal Centre of Tongji Medical College, Huazhong University of Science and Technology. This study was performed in strict accordance with the recommendations in the Guide for the Care and Use of Laboratory Animals of the National Institutes of Health. The protocol was approved by the Institute Animal Care and Use Committee at Tongji Medical College, Huazhong University of Science and Technology (Permit Number: IACUC-2010-306). Their care, housing and all manipulations were conducted in accordance with the Guidelines for the Care and Use of Experimental Animals (Animal Care and Use Committee of Tongji Medical College, Wuhan, China). The animal tumor model was generated by subcutaneous injection with 0.2 ml of cell suspension containing 1 × 10^7^ cells into the right flank of nude mice. When tumor reached an average size of 1.0 cm × 1.0 cm, the mice were randomized into different groups and numbered. In addition, all mice were administrated 1% potassium iodide in their drinking water for 1 week before the experiment, in order to reduce thyroid exposure.

The nude mice bearing human liver cancer were randomized into 4 groups (5 mice per group). The first and second groups were intravenously injected with ^131^I-hVEGF siRNA/SilenceMag, the third with hVEGF siRNA/SilenceMag, and the fourth with normal saline as control. An EMF was bound on the tumor in the nude mice of the first and third group. Mice in all groups were injected once a day for 3 days. The weight of mice and the tumor size, including length and width, were recorded every day. After 4 weeks, mice were all sacrificed, and their tumors were immediately harvested for immunohistochemical assay. The curative effect and the toxicity for each treatment strategy were evaluated with reduction of tumor size and loss of body weight, respectively.

The dissected tumor tissues were fixed in 4% paraformaldehyde for 12 h, embedded in paraffin, and serially sectioned in 4 μm thickness. The tissue was then incubated with VEGF (C-1), a mouse monoclonal antibody (Mitaka Biotechnology Co., Ltd) (1:50 dilution, Santa Cruz Biotechnology, Santa Cruz, USA) overnight at 4°C, and labeled with Streptavidin-Biotin Complex for detection. The slides were counterstained in hematoxylin, dehydrated through graded ethanol solutions and cover-slipped.

### Blood kinetics of ^131^I-labeled hVEGF siRNA/SilenceMag

Six nude mice bearing human liver cancer were intravenously injected with ^131^I-hVEGF siRNA/SilenceMag (3.7 MBq/0.2 ml). Three nude mice were selected at random. An external magnetic field (EMF) with magnetic flux density of 0.8 T was positioned at the tumor surface region on each mouse and remained attached for 12 h. Approximately 20 ul of blood from tail vein was collected in capillary tubes at 0.5 h、1.0 h、1.5 h、2.0 h、3.0 h、4.0 h、6.0 h、8.0 h、10.0 h and 12.0 h after tracer injection. Activity in each blood sample was measured in a γ-counter, and expressed as a percentage of injected doses per gram of tissue (% ID/g).

### SPECT study and biodistribution of ^131^I-hVEGF siRNA/SilenceMag

Six tumor-bearing nude mice, randomly divided into two groups, were injected intravenously with 18.5 MBq/0.2 ml ^131^I-hVEGF siRNA. An EMF was bound on the tumor of each mouse from one group. All the mice were subjected to SPECT imaging (GE Infinia Hawkeye4) at 30 min after tracer injection. The mice were then sacrificed for biodistribution study. Tumors and the following tissues were removed and washed: heart, lung, muscle, bone, liver, kidney, spleen, small intestine, and stomach. Then, the dissected tissues were assayed in a γ-counter. The results were expressed as % ID/g.

### MRI study

Another six tumor-bearing nude mice were intravenously injected with ^131^I-hVEGF siRNA/SilenceMag (18.5 MBq/0.2 ml). Three nude mice were selected at random, and an EMF with a magnetic flux density of 0.8 T remained on tumor surface region for 30 min in each mice. All the mice underwent a T1WI/T2WI MRI scan (3.0 T MRI system, Siemens Magnetom Trio) at 30 min after tracer injection (T1WI: TR/TE 360/13.7 ms, 320 × 320 matrix, FOV 60 × 60 mm^2^, 140 Hz/Px of bandwidth, 1.5 mm slice thickness; T2WI: TR/TE1980/133.9 ms, 320 × 320 matrix, FOV 60 × 60 mm^2^, 220 Hz/Px of bandwidth, 1.5 mm slice thickness). Then, the mice were sacrificed for the biodistribution study with the above-mentioned method.

### Statistical analysis

Group data are expressed as the mean ± SD. Nonparametric Tests were used for data analysis. A P value of ≤0.05 indicate statistical significance. Statistical calculations were performed using SPSS 18.0 for Windows (SPSS, Chicago, IL, USA).

## Results

### ^131^I-Labeled hVEGF siRNA/SilenceMag obtained showed satisfactory radiochemical purity

The ^131^I-labeled siRNA was successfully prepared using Bolton-Hunter reagent (Figure 1A). The recovery rate of hVEGF siRNA was >93.80% after ultrafiltration concentration. The ^131^I-labeled hVEGF siRNA had a radiochemical yield of 18.50 ± 4.80% and a specific activity of about ~2.20 × 10^6^ Bq/nmol.

The ITLC curves of the concentrated ^*131*^*I-*Labeled hVEGF siRNA/SilenceMag samples showed a single peak, demonstrating that the ^131^I-labeled Bolton-Hunter reagent and free ^131^I were effectively removed through ultrafiltration. Further analysis showed that the radiochemical purity of the product was >80% (80.46% from the gel plate chromatography, 84.05% from No. 1 chromatography filter paper).

### HVEGF siRNA/SilenceMag effectively knock down VEGF expression in HepG2 cells

To test the biological activity of hVEGF siRNA, we incubated HepG2 cells with ^131^I-hVEGF siRNA/SilenceMag in a concentration similar to that *in vivo*. As shown in Figure 1B, both hVEGF siRNA/SilenceMag and ^131^I-hVEGF siRNA/SilenceMag caused hVEGF mRNA cleavage effectively in HepG2 cells. hVEGF expression was more than 5 folds lower compared with non-transfected (a) and SiScr (b) by qRT-PCR analysis (Figure 1B). No significant difference in the relative levels of hVEGF mRNA was observed among cells transfected with hVEGF siRNA/SilenceMag and ^131^I-hVEGF siRNA/SilenceMag (*t* = 0.21, *P* = 0.85).

### Blood clearance and biodistribution analysis of ^131^I-labeled hVEGF siRNA/SilenceMag

In order to explore whether ^131^I- hVEGF siRNA/SilenceMag administrated intravenously can reach the tumors and test its distribution in the tumor-bearing nude mice, we measured the kinetics of its blood clearance and its biodistribution in the nude mice bearing human liver cancer. The clearance of ^131^I-hVEGF siRNA/SilenceMag in blood followed a monoexponential elimination pattern and remained in the bloodstream for a short time. The average biologic half-life (T_1/2_) was calculated: 2.22 ± 0.17 h for the mice in the presence of EMF, and 3.37 ±1.17 h for the other mice in the absence of EMF. No significant difference between the two groups was found (Z = 1.96, *P* = 0.05).

**Figure 2:**
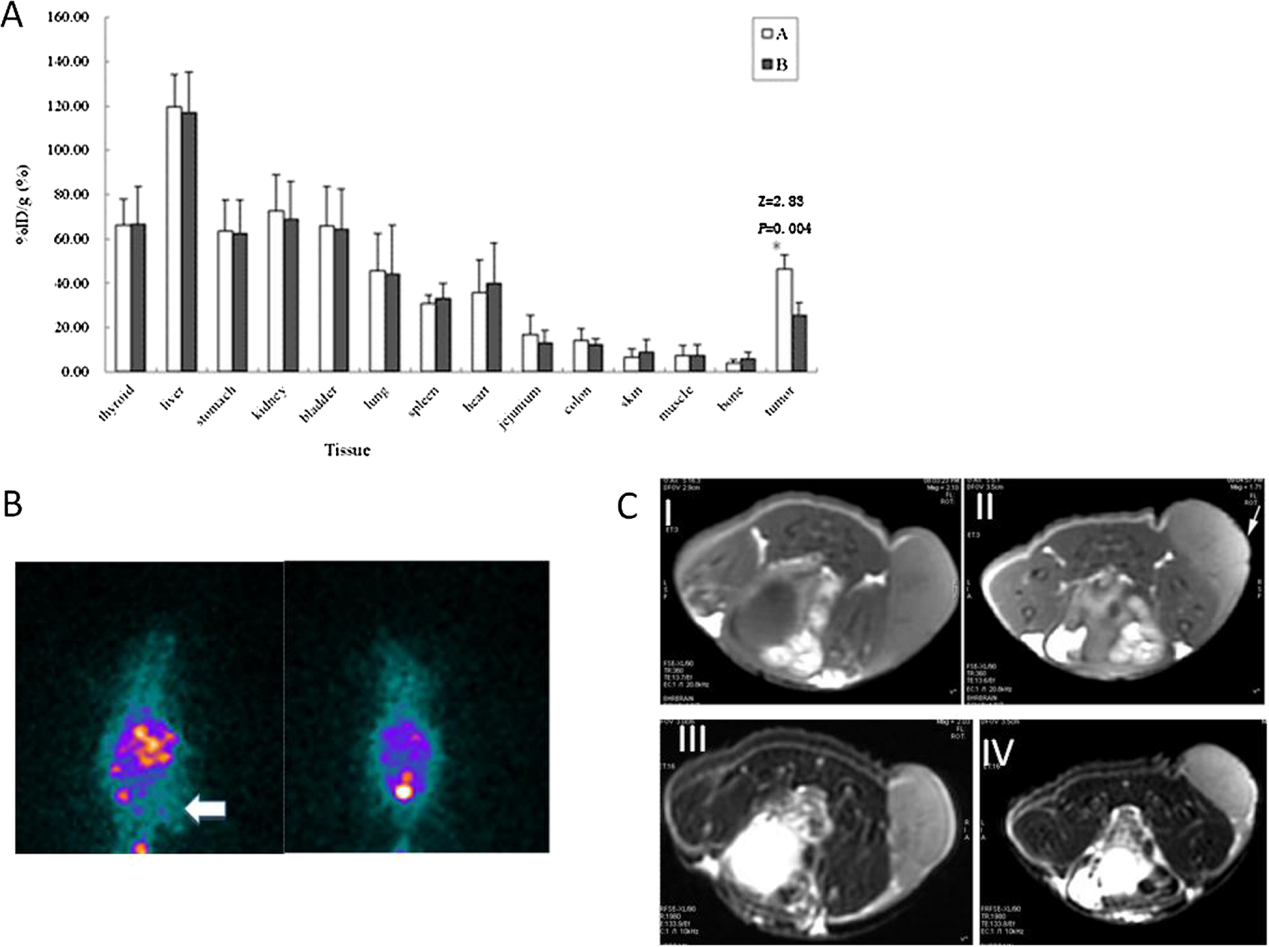
**EMF guidance caused more accumulation and retention of **^**131**^**I-hVEGF siRNA/SilenceMag and hVEGF siRNA/SilenceMag in the HCC tumor compared with no EMF and control. A**: The biodistribution of intravenous 131I-hVEGF siRNA/SilenceMag in HCC tumors as well as different organs 30 min after administered to HepG2 xenografts in the presence of EMF **(A)** or in the absence of EMF **(B)**. **B**: ^131^I-hVEGF siRNA/SilenceMag scintigraphy taken at 30 min after injection in the HepG2 HCC xenograft. Left: in the presence of EMF. Right: in the absence of EMF. **C**: MRI images of nude mice bearing the HepG2 HCC xenograft 30 min after intravenously injection of 131I-hVEGF siRNA/SilenceMag. Upper (T1WI): I, in the absence of EMF. II, in the presence of EMF. Lower (T2WI): III, in the absence of EMF. IV, in the presence of EMF.

Our result showed that the%ID/g of ^131^I-hVEGF siRNA/SilenceMag in tumors with an EMF was more than 2 folds higher than the groups without an EMF (Z = 2.83, *P* = 0.004). And the % ID/g of ^131^I-hVEGF siRNA/SilenceMag in all other collected organs did not show significant differences with or without an EMF (Figure 2A).

### ^131^I-Labeled hVEGF siRNA/SilenceMag accumulated more in HCC tumor guided by EMF

SPECT study showed that there was increased uptake of ^131^I-hVEGF siRNA/SilenceMag in the tumors in the ^131^I-hVEGF siRNA/SilenceMag group in the presence of an EMF compared with the group without an EMF (Figure 2B). Mildly increased signal intensity was found on T1WI MRI on the periphery of tumors in the group of ^131^I-hVEGF siRNA/SilenceMag in the presence of EMF compared with those in the absence of EMF (Figure 2C). However, there was no significant hypointense on the tumor lesions of both groups on T2WI MRI (Figure 2C).

### EMF-guided ^131^I hVEGF siRNA/SilenceMag inhibited HCC tumor growth in nude mice without causing significant weight loss

**Figure 3:**
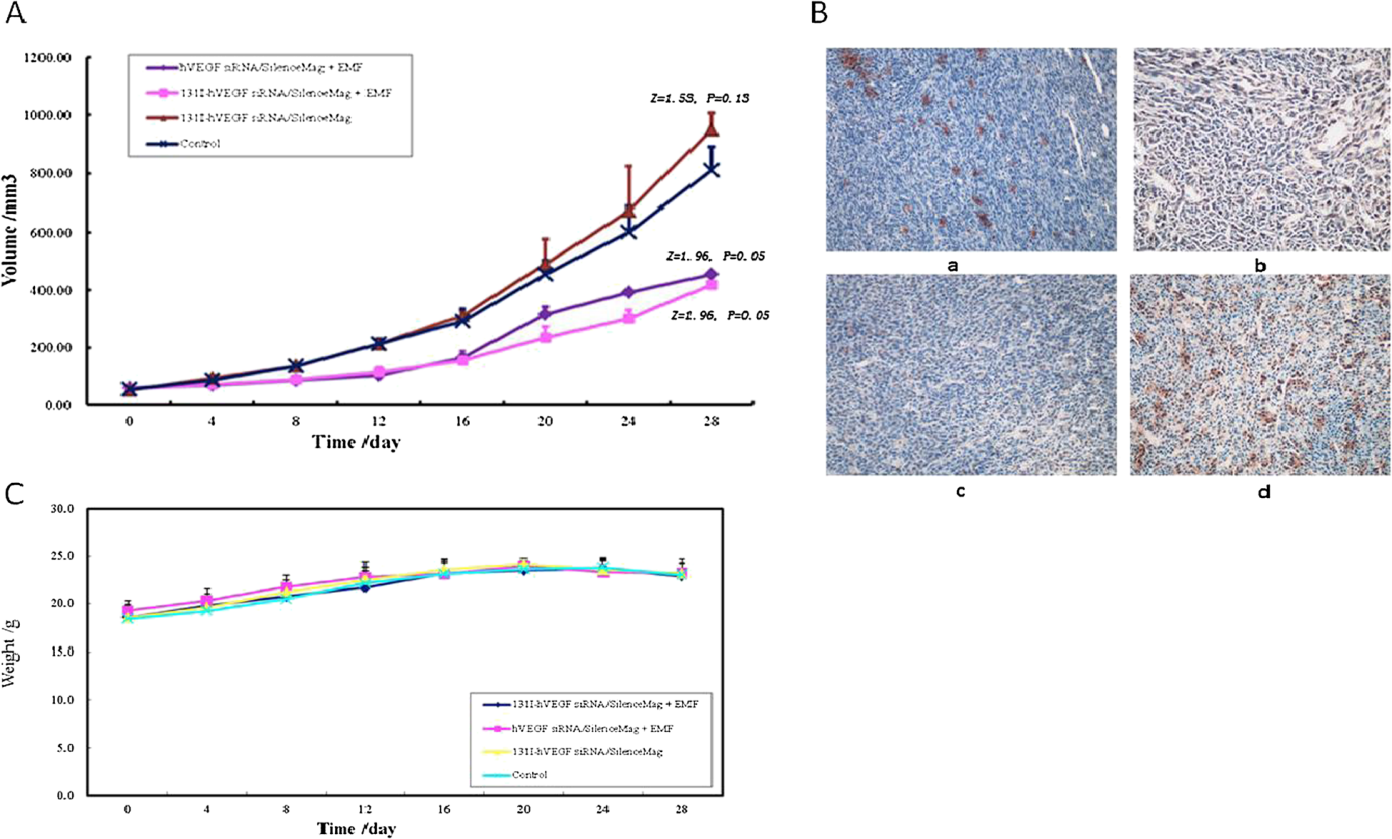
**hVEGF siRNA/SilenceMag and **^**131**^**I-hVEGF siRNA/SilenceMag administration guided by an EMF inhibited HCC tumor growth in nude mice. A**: tumor growth curve after. hVEGF siRNA/SilenceMag and ^131^I-hVEGF siRNA/SilenceMag xenograft. (1) ^131^I-hVEGF siRNA/SilenceMag with EMF; (2) ^131^I-hVEGF siRNA/SilenceMag without EMF; (3) hVEGF siRNA/SilenceMag with EMF; (4) control group with saline solution. **B**: VEGF protein expression by immunohistochemical assay in nude mice bearing HepG2 xenografts 28 days post siRNA administration (magnification × 200). VEGF protein shows as brownish staining. **(a) **^131^I-hVEGF siRNA/SilenceMag administered intravenously guided by EMF; **(b) **^131^I-hVEGF siRNA/SilenceMag administered intravenously in the absence of EMF; **(c)** hVEGF siRNA/SilenceMag administered intravenously guided by EMF; **(d)** the control group. **C**: The weight log of the nude mice bearing the HepG2 HCC xenograft from day 0, injection of hVEGF siRNA/SilenceMag, to day 28. (1) ^131^I-hVEGF siRNA/SilenceMag with EMF; (2) ^131^I-hVEGF siRNA/SilenceMag without EMF; (3) hVEGF siRNA/SilenceMag with EMF; (4) control group.

The tumor growth curves in different groups were shown in Figure 3A, and the tumor weight and tumor inhibition rate were listed in Table 1. The final weight of the tumor in the hVEGF siRNA/SilenceMag group and ^131^I-hVEGF siRNA/SilenceMag group with EMF were less than that of the ^131^I-hVEGF siRNA/SilenceMag group without EMF and the control group four weeks after the treatment. The inhibition rate of ^131^I-hVEGF siRNA/SilenceMag was 49.80% compared with control while there was no significant difference in tumor growth between the hVEGF siRNA/SilenceMag and the ^131^I-hVEGF siRNA/SilenceMag groups. Immunohistochemical assay showed a dramatically decreased expression of VEGF protein in the tumors of hVEGF siRNA/SilenceMag and ^131^I-hVEGF siRNA/SilenceMag groups with EMF, whereas the tumors of ^131^I-hVEGF siRNA/SilenceMag without EMF and the control group showed a higher VEGF protein expression (Figure 3B).

**Table 1:** **Tumor inhibition rate of **^**131**^**I-hVEGF siRNA/SilenceMag 28 days post treatment**

**Group**	**Tumor weight (mg)**	**Tumor inhibition rate (%)**
	**(**$\bar{x}$**±s)**	
^131^I-hVEGF siRNA/SilenceMag + EMF	460.10±29.50	49.80±3.25
	(Z=1.96, *P*=0.05)^*^	
^131^I-hVEGF siRNA/SilenceMag	978.20±19.90	-6.70±2.20
	(Z=1.09, *P*=0.26)^*^	(Z=1.96, *P*=0.05)^#^
hVEGF siRNA/SilenceMag + EMF	499.70±7.60	48.10±5.30
	(Z=1.96, *P*=0.05)^*^	(Z=0.66, *P*=0.51)^#^
Control	916.60±91.20	―

Z-Value was placed within bracket. *Compared with control. ^#^Compared with the first group (^131^I-hVEGF siRNA/SilenceMag + EMF).

The weight log of the nude mice bearing the HepG2 HCC xenograft from different groups was shown in Figure 3C. There was no significant difference in weight loss among four groups for the duration of the 4-week treatment (X^2^_r_ = 0.93, *P* = 0.82). This result indicated that the dosage of ^131^I-hVEGF siRNA/SilenceMag administrated in this study did not cause significant cytotoxicity.

## Discussion

In the search for a novel and integrative therapeutic approach in the treatment of HCC, we employed ^131^I-hVEGF siRNA/SilenceMag, a type of SPIOs that carries hVEGF siRNA and radiolabeled I^131^. VEGF siRNA is unstable and has a short half-life. This problem can be overcome by chemical modification of the synthesized siRNA. We chose 2′-*O*-methyl [18,19] for the modification of our siRNA instead of phosphorothioate [20]. VEGF siRNA activity decreases as the number of 2′-*O*-methyl modified nucleotides increases [21]. We modified three bases at the 5′ ends of each of the two chains. In this study, the sense strand of our precursor hVEGF siRNA was modified with a 5′ amino C6 linker in order to react with *N*-hydroxysuccinimide esters of Bolton-Hunter reagent [22] for formation of stable amide bonds. Then the ^131^I-hVEGF siRNA was successfully prepared using Bolton-Hunter reagent. The hVEGF siRNA recovery rate was high. Its radiochemical yield was 18.5 ± 4.8% and the specific activity was about ~2.22 × 10^6^ Bq/nmol. In addition, SilenceMag retains its biological activity and can deliver hVEGF siRNA inside HepG2 cells. During the entire process of the labeling reaction, SilenceMag did not affect the biological activity of hVEGF siRNA, which is a prerequisite condition for gene therapy.

Our biodistribution analysis indicated that ^131^I-hVEGF siRNA/SilenceMag stayed in the bloodstream for a short time. (T_1/2_: 2.22 ± 0.17 h in the presence of EMF, 3.37 ± 1.17 h in the absence of EMF). ^131^I-hVEGF siRNA/SilenceMag can be a tracer in the SPECT study and the biodistribution study. Both studies showed the increased uptake of ^131^I-hVEGF siRNA/SilenceMag in the tumors of nude mice exposed to EMF. This result confirmed the efficacy of SilenceMag as the carrier of ^131^I-hVEGF siRNA under the influence of an EMF in vivo. Additionally, because of the enhanced permeability and retention effect in tumor vasculature [23,24], ^131^I-hVEGF siRNA/SilenceMag gathering around the tumors was more easily extravasated in tumor tissues for target gene silencing. We also observed that ^131^I-hVEGF siRNA/SilenceMag accumulated slightly more in some normal tissues, such as liver, spleen, stomach, thyroid, kidneys, bladder etc. The reason may be as follows: ^131^I-hVEGF siRNA/SilenceMag cannot evade the phagocytic uptake by the reticuloendothelial system of the liver and spleen [25,26]; the end-product of ^131^I-hVEGF siRNA/SilenceMag metabolism in nude mice was excreted by the kidneys; ^131^I-hVEGF siRNA/SilenceMag may break down in the body and free ^131^I could accumulate in the thyroid and stomach.

We also attempted to use MRI to localize ^131^I- hVEGF siRNA/SilenceMag in the tumors. Mildly increased signal was found on T1WI MRI on the periphery of tumors in the group of ^131^I-hVEGF siRNA/SilenceMag with EMF compared to that without EMF. The T1 relaxation process requires close proximity of the hydrogen atoms to the contrast agent [25,27,28], it appears that SilenceMag has a good hydrophilic nature and is in close proximity to water molecules, leading to shortening of the spinlattice relaxation time and an increased signal intensity. However, there was no significant hypointense signal on the tumor lesions on T2WI MRI, which is different from previous reports [27]. This could be due to the different characteristics of SPIOs and the limited amount of SilenceMag in the local tumor microenvironment. The T2 relaxation occurs because of the exchange of energy between protons in water molecules [25,27,28]. In the presence of EMF, SPIOs create inhomogeneity in the magnetic field of the microenvironment which leads to dephasing of the magnetic moments of protons and hence caused T2 shortening. In this study, the amount of ^131^I-hVEGF siRNA/SilenceMag might be too limited to shorten T2 thus appear hypointense. Therefore, we concluded that ^131^I-hVEGF siRNA/SilenceMag is less sensitive in MRI study than in SPECT imaging.

We successfully validated the antitumor efficacy of hVEGF siRNA/SilenceMag in xenograft nude mice using intravenous injection. The tumor-bearing nude mice receiving ^131^I-hVEGF siRNA/SilenceMag and hVEGF siRNA/SilenceMag with EMF showed a considerable reduction of tumor growth compared to those receiving ^131^I-hVEGF siRNA/SilenceMag without EMF and the controls. However, there was no significant difference in tumor growth between the ^131^I-hVEGF siRNA/SilenceMag group and the hVEGF siRNA/SilenceMag group in the present of EMF. This may be caused by the limited dosage of ^131^I radiation in the local tumors. The results from immunohistochemical assay demonstrated a strong inhibitory effect of VEGF siRNA on VEGF expression in the tumors of the groups receiving ^131^I-hVEGF siRNA/SilenceMag and hVEGF siRNA/SilenceMag with EMF. Finally, there was no significant difference in weight loss among the four groups for the 4-week duration of the siRNA treatment, which suggests that ^131^I-hVEGF siRNA/SilenceMag t has a tolerable cytotoxicity.

Our study here suggests that SilenceMag mediated ^131^I-hVEGF siRNA guided by EMF for HCC targeted therapy has potential prospective. However, a few problems need to be addressed in future research: (1) the preparation of ^131^I-hVEGF siRNA was simple in this study, but it had a relatively low radiochemical yield (18.5 ± 4.8%), which went against synergic hVEGF siRNA gene therapy and ^131^I brachytherapy; (2) ^131^I-hVEGF siRNA/SilenceMag need to be guided by a magnetic field for targeting tumors, which limit its application; (3) the accumulation of ^131^I-hVEGF siRNA/SilenceMag in non-targeted tissues may induce adverse effects in vivo. Future studies will focus on the improvement of the labeling method of ^131^I-hVEGF siRNA and suitable surface modification on SilenceMag to improve its stability and increase its targeted function in vivo.

## Conclusions

Radioiodine labeling of hVEGF siRNA by the Bolton-Hunter method is simple and reliable, although its radiochemical yield needs further improvement. SilenceMag can successfully deliver hVEGF siRNA into the tumors, guided by an EMF, with angiostatic and antitumoral effects via VEGF knock-down. The dual functional properties of ^131^I-hVEGF siRNA/SilenceMag in tumor therapy and imaging provide an attractive system where real time monitoring of gene delivery, gene therapy and radionuclide brachytherapy can be integrated. Therefore, the synergy therapy of ^131^I-hVEGF siRNA/SilenceMag might be a future treatment option against HCC.

## Competing interests

The authors declare that they have no competing interests.

## Authors’ contributions

CJ and XW conceived and designed the study. ZS, TLQ, LJS, ZM, CJ conducted the experiments. ZS, TLQ, LJS, ZM, CJ analyzed and interpreted the data. The manuscript was drafted by CJ and ZS and critical revision was done by CJ. All authors read and approved the final manuscript.

## Pre-publication history

The pre-publication history for this paper can be accessed here:

http://www.biomedcentral.com/1471-2407/14/114/prepub
